# Digestive Duet: Midgut Digestive Proteinases of *Manduca sexta* Ingesting *Nicotiana attenuata* with Manipulated Trypsin Proteinase Inhibitor Expression

**DOI:** 10.1371/journal.pone.0002008

**Published:** 2008-04-23

**Authors:** Jorge A. Zavala, Ashok P. Giri, Maarten A. Jongsma, Ian T. Baldwin

**Affiliations:** 1 Department of Molecular Ecology, Max Planck Institute for Chemical Ecology, Jena, Germany; 2 Plant Molecular Biology Unit, Division of Biochemical Sciences, National Chemical Laboratory, Pune, India; 3 Plant Research International B.V., Wageningen University and Research Centre (WageningenUR), Wageningen, The Netherlands; Massachusetts General Hospital, United States of America

## Abstract

**Background:**

The defensive effect of endogenous trypsin proteinase inhibitors (NaTPIs) on the herbivore *Manduca sexta* was demonstrated by genetically altering NaTPI production in *M. sexta's* host plant, *Nicotiana attenuata.* To understand how this defense works, we studied the effects of NaTPI on *M. sexta* gut proteinase activity levels in different larval instars of caterpillars feeding freely on untransformed and transformed plants.

**Methodology/ Principal Findings:**

Second and third instars larvae that fed on NaTPI-producing (WT) genotypes were lighter and had less gut proteinase activity compared to those that fed on genotypes with either little or no NaTPI activity. Unexpectedly, NaTPI activity *in vitro* assays not only inhibited the trypsin sensitive fraction of gut proteinase activity but also halved the NaTPI-insensitive fraction in third-instar larvae. Unable to degrade NaTPI, larvae apparently lacked the means to adapt to NaTPI in their diet. However, caterpillars recovered at least part of their gut proteinase activity when they were transferred from NaTPI-producing host plants to NaTPI-free host plants. In addition extracts of basal leaves inhibited more gut proteinase activity than did extracts of middle stem leaves with the same protein content.

**Conclusions/ Significance:**

Although larvae can minimize the effects of high NaTPI levels by feeding on leaves with high protein and low NaTPI activity, the host plant's endogenous NaTPIs remain an effective defense against *M. sexta*, inhibiting gut proteinase and affecting larval performance.

## Introduction

Although phosphorous has recently been proposed to be very important for insects in certain environments [1], nitrogen (N) is thought to be the main nutrient for Lepidoptera (e.g., [2]–[5]). Since proteins are the main source of N for insects, and their quality and quantity change within the plant, it is not surprising that larvae exhibit complex resource-oriented foraging behaviors that change with their changing dietary needs and development [3], [6]–[8]. Plants respond to insect attack by producing defensive proteins, such as proteinase inhibitors (PIs), that inhibit protein utilization and decrease larval performance 9–11.

Plant PI proteins elicited in response to insect attack affect the digestibility of ingested proteins, decreasing the amount of free amino acids required for growth, development, and reproduction [12]. Since insects are unable to synthesize a number of amino acids, they depend on digestive proteases and plant proteins to meet their nutritional requirements [13], [14]. Serine proteinases are common in the alkaline midgut of lepidopteran larvae, and most species examined contain proteinases with different specificity, such as trypsin-like and chymotrypsin-like enzymes [15]–[17]. Although PIs can be an effective defense against herbivores, reducing larval survivorship and growth rate, larvae can adapt to high PI levels in the plant species that they feed on [18].

Insects are known to respond to high PI levels in their food by increasing consumption [19], [20] and/or by changing the molecular and biochemical environment of their guts [21], [22]. The most widespread strategies insects use to counter PIs is to produce proteases that are insensitive to the inhibitor [23]–[25] and/or to proteolytically inactivate PIs with midgut proteases [26], [27]. Evidence for the effects of PIs on gut proteinases comes from experiments with insects that fed on plants heterologously expressing *pi* genes or artificial diets containing PIs; no study to date has altered the expression of an endogenous *pi* gene in a host plant to examine its effect on lepidopteran digestive enzymes.

Studies with artificial diets provide a valuable way to manipulate the ingestion of PIs independently of the ingestion of protein [28]; however, these diets frequently contain proteins such as casein, wheat germ, or seed powder, which are not natural and lack the complement of other phytochemicals normally found in plant tissues [21], [29]–[31]. These drawbacks are overcome in studies with plants that express a novel *pi* gene without altering the expression of other phytochemicals (e.g.,[29], [32], [33]). However, in the process of adapting to a particular host plant, insects may evolve gut proteinases that are resistant to the PIs of their hosts [34]–[37]. Hence, heterologous expression studies with constitutive promoters do not reflect the dynamics that are likely to occur in natural plant-insect interactions (e.g., [29], [38]).

*Nicotiana attenuata* Torr. Ex Wats., a post-fire annual inhabiting the Great Basin Desert, has a number of well-described herbivore-induced direct and indirect defenses [39]. In addition to nicotine, *N. attenuata* produces trypsin proteinase inhibitors (NaTPIs), which reduce the performance of herbivores [40]. Although constitutive and inducible NaTPI expression in *N. attenuata* is costly when plants are not attacked, resulting in reduced seed capsule production and plant growth [40], the fitness costs of NaTPI expression are balanced by its fitness benefits when plants are attacked by the natural herbivore *Manduca sexta* [41]. Adult female *M. sexta* typically oviposit on the basal rosette leaves of *N. attenuata* in their native habitats. After the larvae begin to feed on these leaves, the plants respond with local and systemic increases in NaTPI levels and with post-translational changes of the NaTPI precursor, increasing the structural diversity of the NaTPI isoinhibitors [42]–[44]. Larvae tend to remain on the leaf on which they hatched during the first instar, but between second and third instars they often leave this highly elicited leaf and move upward within the plant to feed on young leaves, which have higher levels of protein and lower levels of NaTPI activity (i.e., a low NaTPI: protein ratio). Such a change in feeding location increases larval mass and decreases plant fitness [41], [45]. Sequences of trypsin and one chymotrypsin-like cDNA have been identified in the midguts of *M. sexta* larvae [46], [47]. The larvae may alter the expression of these proteinases to compensate for the ingestion of NaTPIs, as has been described for other inhibitors and lepidoptera [21], [48].

By genetically modifying the ability of *N. attenuata* to produce NaTPI, we were able to address the following questions: Can the growth reductions observed in *M. sexta* larvae feeding on NaTPI-containing plants be attributed to the inhibition of gut proteinases? Do larvae adapt to dietary NaTPIs when they feed freely on plants? Does the NaTPI:protein ratio in the diet influence insect growth and digestive proteinase activity? How does the elicitation of plants by feeding insects affect the adaptation of digestive proteases to dietary TPIs? Since lepidopteran larvae can readjust both their metabolism and feeding behavior to cope with PI intake [6], [8], [24], [49], we determined the effects of an endogenous PI on performance and gut proteases of different larval instars while larvae fed at their natural feeding positions on plants; such positions differed only in the expression of an endogenous *pi* gene. Ideally, the defense function of endogenous *pi* can be determined in plants that differ only in a gene that controls the expression of a resistance trait but are otherwise identical [50].

## Materials and Methods

### *N. attenuata* genotypes and growth conditions

**Figure 1:**
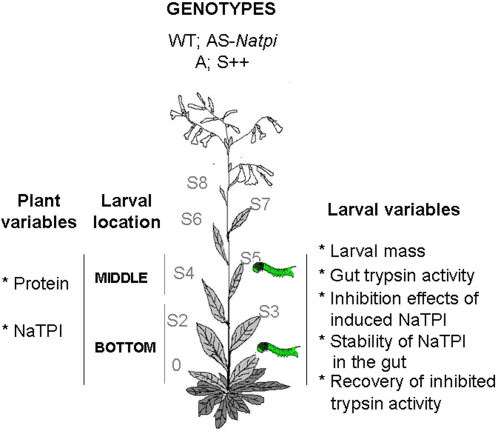
Outline of the different either larval or plant variables measured in this study. Four *N. attenuata* genotypes were used: untransformed wild type (WT) plants of the Utah genotype (WT); a homozygous T_3_ line of the WT genotype transformed with a construct containing a 175 bp *pi* gene fragment in an anti-sense orientation (AS-*Natpi*); untransformed plants of the Arizona (A) genotype; or plants of the Arizona genotype transformed with a construct containing the full-length *pi* gene in a sense orientation (S++). Two plant variables were measured at either the BOTTOM (S1) or the MIDDLE (S5) of plants: leaf protein content and NaTPI activity. *M. sexta* neonates were placed either at the MIDDLE or the BOTTOM of plants and five larval variables were measured: caterpillar mass, gut trypsin activity, effects of induced NaTPI on gut trypsin activity, stability of NaTPI in the gut and the recovery of inhibited trypsin activity after larvae moved to plants without NaTPI.

*N. attenuata* used in this study were grown from seeds collected from either Utah [51] or Arizona [52] and inbred 10 and 4 generations, respectively (Fig. 1). In order to silence the expression of *N. attenuata*'s *tpi* (*Natpi*) gene in the genotype collected in Utah (WT), WT plants were transformed by an *Agrobacterium*-mediated transformation procedure with pNATPI1, which harbored 175 bp of *N. attenuata*'s 7-repeat domain *tpi* gene in an anti-sense orientation (AS-*Natpi*; line number A315; Fig. 1), as described in [53]. Southern gel blot analysis confirmed that all T_3_ lines were single-copy independent transformants and all have been fully characterized [40], [53].

In addition, we used a genotype of *N. attenuata* collected from Arizona (A), whose methyl jasmonate (MeJA)-inducible nicotine levels were comparable to those found in WT plants but which completely lacked the ability to produce NaTPI or accumulate NaTPI mRNA [52]. Plants of the A genotype were transformed with a binary transformation vector pRESC2PIA2 containing the full-length 7-domain *Natpi* gene from the WT genotype in the sense orientation under control of the constitutive CaMV 35S promoter [53]. A T_3_ line harboring a single copy of the transgene (S++; line number A981; Fig. 1) with 60% of the activity of MeJA-elicited WT plants was selected for the study [53]. Non-transformed Arizona plants (A) had no detectable trypsin inhibitory activity. Flow cytometric analysis confirmed that all plants were diploid [54].

This is an ideal system in which to examine the defensive function of TPI expression because the transformed (S++ and AS-*Natpi*) and untransformed genotypes (A and WT) did not differ in any other measured defense traits [53]. This similarity allowed us to examine the defensive function of TPIs by constraining plant responses to herbivore attack and to observe unconstrained herbivore behavior in response to these constrained plant responses. In this way, the dynamics of the plant responses, or the lack thereof, are reflected in the herbivores' behavior and their gut protease activity.

Seeds were germinated in diluted liquid smoke solutions as described in [55] and seedlings were transplanted into 1L pots containing 95% peat and 5% clay in a glasshouse under the conditions described in [53] with 1000–1300 µmol m^−2^ s^−1^ PPFD supplied by 450 W Na-vapor HID bulbs. The soil used in our experiments contained the following nutrient concentrations: N 70 mg/L, P 80 mg/L and K 90 mg/L at 5.5–60 pH.

### *M. sexta* larval feeding experiments

In order to determine the effects of either down-regulation or restored expression of the *Natpi* gene in *N. attenuata* on larval mass gain and gut proteinase activity of the native herbivore *M. sexta*, a single neonate was placed on the leaf growing at either node S1 (BOTTOM) or node S5 (MIDDLE) (described in [41] of 24 plants grown in 1L pots of transformed (AS-*Natpi* and S++) and untransformed (WT and A) genotypes (Fig. 1). Eggs of *M. sexta* larvae (L) were obtained from Carolina Biological Supply Company (Burlington, NC, USA) and placed in plastic containers (200 mL) on a moist tissue. The containers were kept in climate chambers at 28°C and 65% relative humidity under a 16:8 h light:dark photoperiod until the eggs hatched. To control for the inhibitory effects of NaTPI on gut proteinase activity, another group of *M. sexta* larvae was reared on artificial diet without PIs, based on casein and wheat germ [56]. Larval mass of 20 caterpillars was determined 7 days after hatching; 3 days later (10 days after hatching) third-instar larvae from the BOTTOM of the plant were dissected and gut proteinase activity was measured. We did not dissect larvae from the MIDDLE of the plant because after approximately day 7, larvae from the BOTTOM of the plant often began to eat leaves at higher stalk nodes on the plant, thereby confounding the effects of leaf position on gut proteinase activity.

The first two instars are known to be the most critical stages for lepidopteran larvae [57]; moreover, *M. sexta* larvae normally do not move among leaves during the first two instars [41]. Therefore, in an additional experiment, we determined the effect of NaTPI on gut proteinases of second-instar larvae that fed at either the BOTTOM or the MIDDLE of the plants. Neonates were placed on transformed (AS-*Natpi* and S++) and untransformed (A and WT) genotypes at either the BOTTOM or the MIDDLE of plants. 50 second-instar (5 days after hatching) larvae were collected and total and gut proteinase activity measured. This experiment was performed twice.

In order to determine the short-term effects of NaTPI on gut proteinases of third-instar larvae adapted to feed on plants either with or without NaTPI, 30 single neonates were placed at the BOTTOM of either A or S++ genotypes. Ten days after hatching, half of the larvae (15) feeding on each genotype were transferred from either A to S++ (A→S++) or S++ to A (S++→A) to the same leaf position at which they had fed on the previous plant, while the other half (15) were picked up and put down on the original feeding positions to control for potential handling effects. After 24h, larvae were collected from all plants and gut proteinase activity levels measured.

### Extraction of *M. sexta* gut proteinases

Midguts, isolated by dissection, were stored at −20°C until needed. Either 5 third- or 10 second-instar larval midguts were pooled and pulverized in liquid nitrogen with a mortar and pestle before being used as a biological replicate. We used at least 4 biological replicates to determine proteinase activity in second- and third-instar caterpillars. Proteinases from midguts were extracted by homogenizing 100 mg of tissue with 200 µl of 0.2 M glycine-NaOH buffer, pH 9.5, and allowing the extract to stand for 15 min at 4°C. The suspension was centrifuged at 10,000 g for 10 min at 4°C and the resulting supernatant used as a source of *M. sexta* gut proteinase activity.

### Proteinase assays

To determine the effects of NaTPI and protein content on the gut proteinase activity of caterpillars that fed at the BOTTOM or MIDDLE of plants, we estimated *in vitro* gut proteinase activity of second- and third-instar larvae. We used azocaseine as a substrate to estimate total protease activity. Sixty µl of 3x diluted enzyme (gut proteinase) was added to 200 µl of 1% azocasein (in 0.2 M glycine-NaOH, pH 9.5) and incubated at 37°C for 30 min. Gut proteinase activity was measured at 13-fold dilution of the *in vivo* concentration. The reaction was terminated by adding 300 µl of 5% trichloroacetic acid. After centrifuging at 10,000 g for 10 min, an equal volume of 1M NaOH was added to the supernatant and absorbance was measured at 450 nm in both samples and controls. One protease unit was defined as the amount of enzyme that increases absorbance by 1 OD/min. Trypsin activity was estimated using the chromogenic substrate, benzoyl-arginyl *p*-nitroanilide (BA*p*NA) [58]; 150 µl of the 3x diluted enzyme was added to 1 ml of 10 mM BA*p*NA (in 0.2 M glycine-NaOH, pH 9.5) and incubated at 37°C for 10 min. Gut trypsin activity was measured at 23-fold dilution of the *in vivo* concentration. The reaction was terminated by adding 200 µl of 30% acetic acid and absorbance measured at 410 nm in both samples and controls. One trypsin activity unit (TAU) was defined as the number of enzymes required to produce 1 mM of 4-nitroaniline per minute at 37°C using BA*p*NA as a substrate under given assay conditions.

### Extraction of leaf proteins and inhibitor assays

Leaves growing at either the BOTTOM or the MIDDLE of the plant were harvested 4 days after neonates started to feed, and soluble protein was extracted as explained in [43]. Protein content and levels of constitutive and induced NaTPI activity resulting from caterpillar damage were determined from 12 replicates. NaTPI activity was determined by radial diffusion assay with bovine trypsin and is expressed as nanomoles per milligram as described in [43].

In order to assess the potentially inhibitory effects of induced NaTPIs on larval proteinase activity and the induction of inhibitor-insensitive proteinase activity in the guts of second- and third-instar larvae that fed at the BOTTOM of plants, we used as inhibitors NaTPI extracts prepared from the BOTTOM of WT plants that were subjected to the following: either uninduced; induced by 4 days of caterpillar attack; or induced after 4 days of wounding followed by the application of *M. sexta* oral secretions to the puncture wounds (W+OS). Proteinases of larvae reared on either WT or artificial diet were used for assays, and the same amount of NaTPI activity (against bovine trypsin) per mg of protein was used for assays to measure gut proteinase activity.

In an additional experiment, we used the protein extracts from either A or S++ genotypes as inhibitors to assess the gut proteinase activity of caterpillars that fed on either A or S++ plants. In these experiments the same proteinase activity was used. Since the extracts used in this experiment were from uninduced A and S++ plants and the only difference between these genotypes is their NaTPI expression, this experiment allowed us to separate the potential effects of compounds other than NaTPI on gut proteinase activity.

### NaTPI stability in relation to gut proteolytic activity

To determine the stability of NaTPIs in relation to gut proteinase activity, leaf extracts containing NaTPI were incubated with gut proteinase (1 TAU) from third-instar caterpillars with 2 trypsin inhibitory units at 30°C for 30 min and 3 h. The mixture was then resolved on a 10% native polyacrylamide gel electrophoresis (PAGE) in a vertical slab gel unit (Hoefer SE 600, Amersham Pharmacia Biotech Inc., Piscataway, NJ, USA) using the Davis buffer system [59]. NaTPIs were visualized using the gel X-ray film contact print technique [60]. After electrophoresis, the gel was equilibrated in 0.1 M Tris-HCl (pH 7.8) twice for 15 min. The gel was then incubated in 0.02% trypsin solution for 15 min, and the excess trypsin was removed by rinsing, after which the gel was overlaid on an X-ray film (Konica X-ray film AX, Goa, India) for 4 and 8 min. The X-ray films were washed with tap water and inhibitor activity bands were visualized as unhydrolyzed gelatin on the X-ray film.

### Statistical analysis

Data were analyzed with SPSS for Windows, Version 14.0 (SPSS Inc, Chicago, IL, USA). NaTPI, protein concentration, larval mass, proteinase activity, and inhibition values were analyzed by ANOVAs followed by Tukey HSD post-hoc comparisons in all experiments. NaTPI extracts at different standardized protein concentrations values were analyzed by repeated measures ANOVA. All proportions were arcsine square-root transformed before statistical analysis to correct non-normality.

## Results

### NaTPI activity in *N. attenuata* genotypes and its effects on *M. sexta* larvae

**Figure 2:**
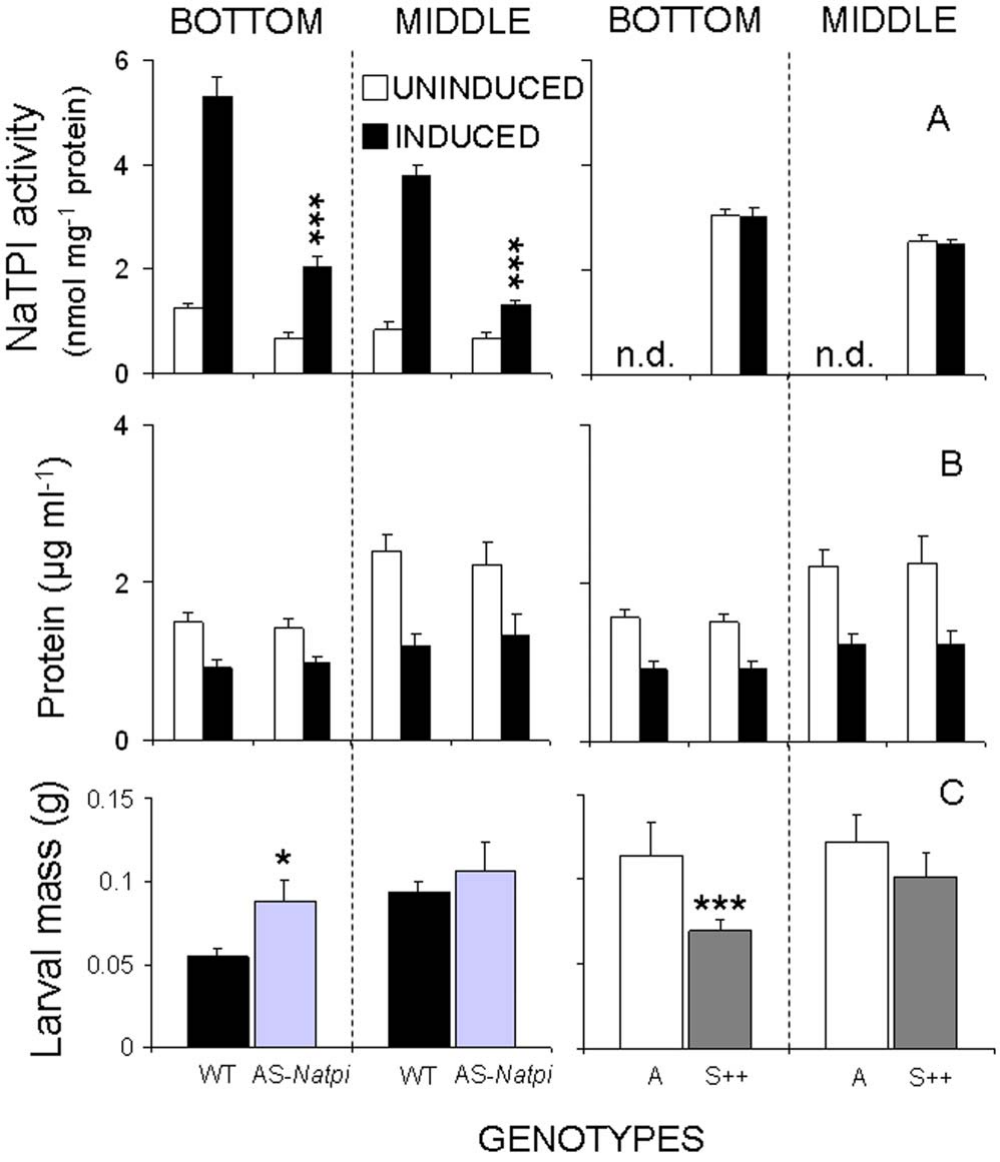
Trypsin proteinase inhibitors (NaTPI) and protein contents of *N. attenuata*. A. NaTPI. B. protein contents (mean 1±SEM) in leaves growing at node S1 (BOTTOM) or node S5 (MIDDLE) which had been either UNINDUCED or INDUCED by caterpillars feeding for 4 days, and C. *M. sexta* larval mass (mean 1±SEM) 7 days after neonates started to feed on leaves at either S1 (BOTTOM) or node S5 (MIDDLE) positions of: WT, AS-*Natpi*, A or S++ plants. n.d. = not detectable in the A genotype. Symbols above columns indicate levels of significant differences with respect to untransformed genotypes either A or WT (*P< 0.05, **P< 0.001, ***P< 0.0001).

The activity levels of *N. attenuata* trypsin proteinase inhibitors (NaTPIs) in both uninduced and induced plants after 4 days of caterpillar attack on NaTPI-producing genotypes (untransformed WT and transformed AS-*Natpi, Natpi* gene expressed in an anti-sense orientation, and S++ containing *Natpi* gene in the sense orientation under control of the constitutive promoter) were lower at the MIDDLE than at the BOTTOM of the plants (F_11,143-Natpi_ = 53.752; P < 0.0001; Fig. 2A). Caterpillar attack elicited higher NaTPI activity levels in WT (4.4-fold at the BOTTOM and 4.7-fold at the MIDDLE) than in AS-*Natpi* genotypes (2.9-fold at the BOTTOM and 1.8-fold at the MIDDLE; P < 0.0001; Fig. 2A).

As expected, caterpillar attack did not affect levels of NaTPI activity either at the BOTTOM (P = 1.0) or the MIDDLE of S++ plants (P = 0.9); levels in these plants remained at approximately 57% of the induced WT genotype at the BOTTOM and 65% at the MIDDLE of plants (P < 0.0001; Fig. 2A). Compared to the uninduced levels of NaTPI activity in WT plants, activity levels in the S++ genotype were approximately 2.5-fold higher at both leaf positions (BOTTOM and MIDDLE) (P < 0.0001; Fig. 2A). Although protein content did not differ significantly among genotypes, protein content was higher at the MIDDLE than at the BOTTOM of plants (P < 0.01; Fig. 2B) and caterpillar attack decreased soluble protein levels up to 50% (F_15,191-Protein = _8.084; P < 0.0001; Fig. 2B).

Endogenous NaTPI reduced caterpillar mass, but high protein content at the MIDDLE of the plant likely diluted NaTPI's effect on larval mass (F_7,159 = _4.453; P < 0.0001; Fig. 2C). Although larval masses of caterpillars that fed at the BOTTOM of plants were higher (38%; P = 0.04) in AS-*Natpi* than in WT genotypes and lower (45%; P = 0.04) in S++ than in the untransformed A genotype (which lacks the ability to produce NaTPI), larval masses did not differ significantly between caterpillars that fed at the MIDDLE of high- and low-NaTPI-producing plants (P = 0.98; P = 0.88; Fig. 2C).

### Effects of NaTPI on gut proteinase activity at different larval instars

**Figure 3:**
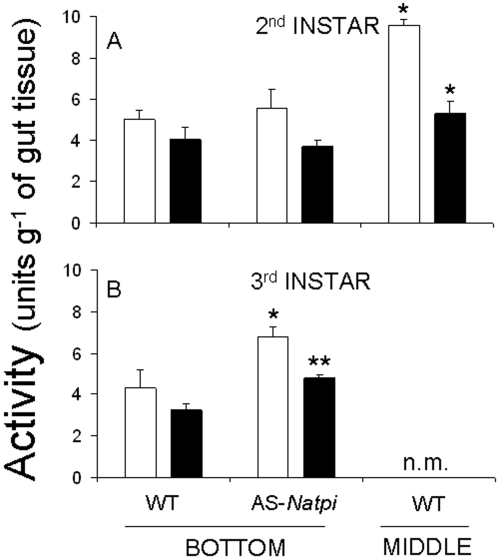
Gut proteinase activity (mean±SEM) of *M. sexta* larvae. Larvae fed on leaves growing at either node S1 (BOTTOM) or S5 (MIDDLE) of WT and AS-*Natpi* genotypes. A. Second-instar caterpillar guts (5 days after hatching). B. Third-instar caterpillar guts (10 days after hatching). Gut proteinase activity levels of third-instar caterpillars that fed at the MIDDLE of plants were not measured (n.m.), as explained in Materials and Methods. Total gut proteinase activity was measured using azocasein as a substrate (black bars), and trypsin-like activity was measured using BA*p*NA as a substrate (open bars). Symbols above columns indicate levels of significant differences between either WT and AS-*Natpi* genotypes at the BOTTOM of plants, or between the BOTTOM and MIDDLE of WT plants (*P< 0.05, **P< 0.001, ***P< 0.0001).

To determine the effects of NaTPI and protein content on the *M. sexta* gut proteinase activity of caterpillars that fed at the BOTTOM or MIDDLE of plants, we estimated gut proteinase activity in second- and third-instar larvae. Second-instar caterpillars (5 days after hatching) that fed at the MIDDLE of WT plants had more gut proteinase activity than those that fed either at the BOTTOM of WT or on AS-*Natpi* plants (F_2,11-AZOCASEIN = _5.20; P = 0.04; F_2,11-BA*p*NA = _7.144; P = 0.02)( Fig. 3A). Moreover, levels of both azocaseinolytic and BA*p*NAase in third-instar caterpillars (10 days after hatching) were higher when larvae fed on AS-*Natpi* than on WT plants (F_1,8-AZOCASEIN = _14.596; P = 0.007; F_1,10-BA*p*NA = _7.189; P = 0.03; Fig. 3B). The gut proteinase activity levels of third-instar caterpillars that fed at the MIDDLE of plants were not measured because larvae from the BOTTOM of the plant often began to eat leaves at higher stalk nodes on the plant, thereby confounding the effects of leaf position on gut proteinase activity.

In summary, high levels of NaTPI activity inhibited the gut proteinase activity of second- and third-instar larvae that fed at the BOTTOM of WT plants. Interestingly, caterpillars that fed at the MIDDLE had higher levels of gut proteinase activity than those that fed at the BOTTOM of plants.

### Consequences of different NaTPI:protein ratios on the inhibition of gut proteinase activity

**Figure 4:**
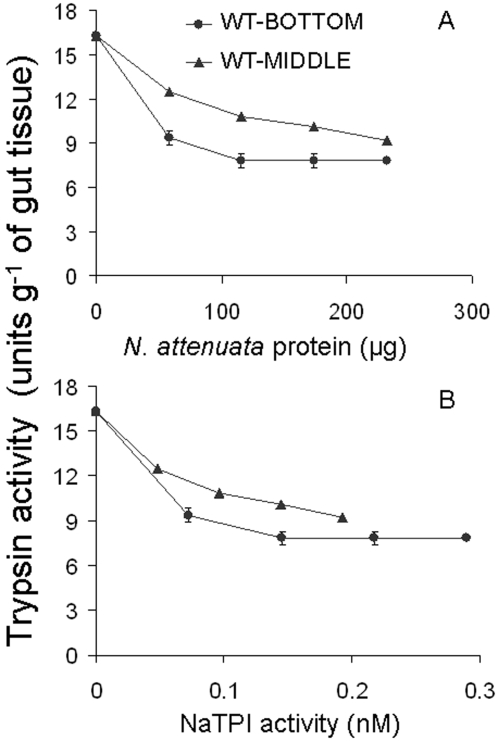
Kinetics of trypsin activities in *M. sexta* guts with different NaTPI levels and protein contents. Larval proteinases were inhibited by adding different dilutions of *N. attenuata*'s trypsin proteinase inhibitor (NaTPI). Either A. different protein concentrations or B. NaTPI activity of WT leaves from either the BOTTOM (•) or the MIDDLE of plants (▴) were used as inhibitors. BA*p*NA was used as a substrate, and the inhibition of hydrolysis of the substrate by gut proteinases after incubation with NaTPI was determined from guts of third-instar caterpillars reared on artificial diet.

Larvae typically move from the older leaves (BOTTOM) with high NaTPI activity and low protein content on which they were oviposited to the younger leaves (MIDDLE) with high protein and low NaTPI activity during second and third instars [41]. We determine the conditions which maximally inhibited the gut proteinases of these highly mobile third-instar caterpillars. We used extracts of older leaves and younger leaves and measured BA*p*NA hydrolysis by gut proteinase. The gut proteinase extracts were incubated with NaTPI extracts at 4 different standardized protein concentrations until maximum trypsin inhibition potential was attained (Fig. 4A). Extracts of basal leaves (WT-BOTTOM) inhibited more gut proteinase activity than did extracts of middle (WT-MIDDLE) stem leaves. In fact, approximately 4 times more protein was required to inhibit the same amount of enzyme activity, a result which is not solely owing to lower NaTPI levels in the middle leaves. When the concentration differences presented in Fig. 4B are taken into account, the NaTPIs extracted from bottom leaves are still about 2.5 times more effective than those from middle leaves (repeated measures ANOVA, F_4,20 = _152.850; P < 0.0001; Fig. 4), suggesting that the TPIs from basal leaves are more efficient at inhibiting gut proteinase than are those from the middle leaves.

### Inhibition effects of induced NaTPI on gut proteinase

**Figure 5:**
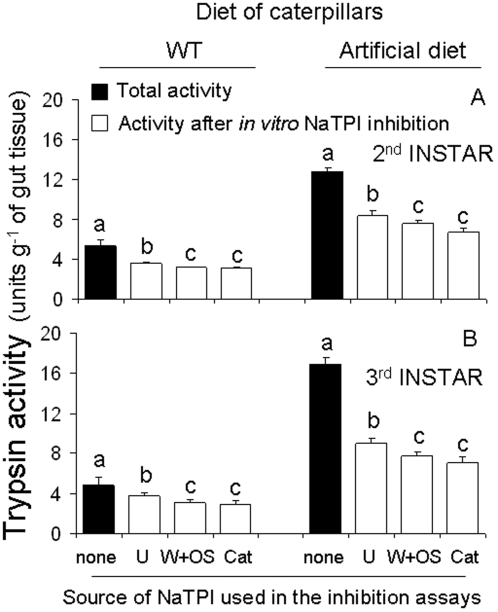
Inhibited digestive trypsin activity (mean±SEM) of *M. sexta* by NaTPI. Trypsin activity was inhibited (open bars) by adding *N. attenuata*'s trypsin proteinase inhibitor (NaTPI). Caterpillars were either reared on artificial diet or fed foliage from WT plants and gut trypsin activity was measured at the following developmental stages: A. second-instar caterpillar guts (6 days after hatching); B. third-instar caterpillar guts (10 days after hatching). NaTPI from the BOTTOM of WT plants either uninduced (U); induced after 4 days of caterpillar attack (Cat); or induced after 4 days of wounding followed by the application of *M. sexta* oral secretions (OS) to puncture wounds (W+OS) were used as the inhibitor in the *in vitro* assays. BA*p*NA was used as a substrate to determine gut proteinase activity. Within each diet of caterpillars and instars, bars with the same letter are not significantly different at P< 0.05.

In order to assess the potentially inhibitory effects of induced NaTPI on proteinase activity in the guts of larvae that fed on plants, we used leaf extracts with the same level of NaTPI activity per mg of protein from the BOTTOM of WT plants; all leaves were subjected to the following: either uninduced (unattacked); induced by 4 days of caterpillar attack; or induced after 4 days of wounding followed by the application of *M. sexta* oral secretions to the puncture wounds (W+OS; Fig. 5). Interestingly, although NaTPI extracts with the same activity per mg of protein were used in the experiments, the effects of extracts of leaves previously elicited by W+OS or caterpillar attack were significantly more inhibitory (38%) than the effects of extracts from uninduced WT plants (22%; P < 0.0001; Fig. 5). Moreover, irrespective of the diet of the caterpillars, NaTPI extracts from induced WT plants inhibited more trypsin activity from the guts of second- (F_5,17-2-INSTAR_ = 21.275; P < 0.0001)( Fig. 5A) and third-instar caterpillars (F_5,17-3-INSTAR = _79.731; P < 0.0001)( Fig. 5B) than those from uninduced WT plants.

### Qualitative and quantitative changes in gut proteinase activity in response to NaTPI expression in the A genotype

**Figure 6:**
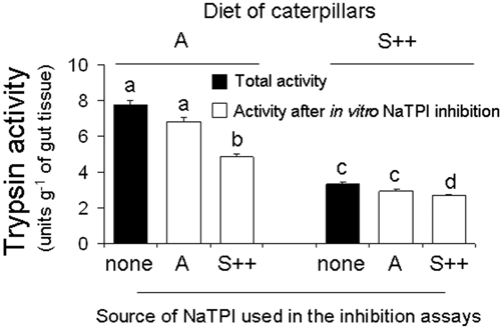
Inhibited trypsin activity (mean±SEM) of *M. sexta* gut proteinases. Trypsin activity was inhibited (open bars) by adding *N. attenuata*'s trypsin proteinase inhibitor (NaTPI). We used the protein extracts from either A or S++ genotypes as inhibitors and BA*p*NA as a substrate in the *in vitro* assays, and determined the inhibition of hydrolysis of the substrate by gut proteinases after incubation with NaTPI. Third-instar caterpillars were fed on leaves growing on either A or S++ genotypes. Bars with the same letter are not significantly different at P< 0.05.

NaTPI produced in the A genotype (S++) strongly inhibited the gut proteinase levels of third-instar larvae (black bars; Fig. 6). Larvae that fed on S++ (3.3 trypsin activity units, TAU) plants had 57 % lower BA*p*NAase activity levels than those that fed on A (7.7 TAU) plants (F_1,5 = _290.829; P < 0.0001; Fig. 6). Similar results were found using azocasein as a substrate (data not shown). Our results suggest that NaTPI expressed in the A genotype is active in the midguts of larvae, which resulted in lower levels of free trypsin-like activity in caterpillars that fed on S++ compared to those that fed on the A genotype.

In order to assess the potentially inhibitory effects of expressed NaTPI on trypsin in the guts of caterpillars that fed on leaves that either contained NaTPI or that did not, we used leaf extracts from either A or S++ genotypes to inhibit a standardized amount of gut proteinase activity. Leaf extracts from the A genotype did not inhibit the gut proteinase activity of caterpillars that fed on either A or S++ genotypes (white bars; P = 0.7).

Leaf extracts from the S++ genotype inhibited almost twice (P < 0.0001) the gut proteinase activity of caterpillars that fed on A (37%; 2.9 TAU) compared to those that fed on S++ (21%; 0.7 TAU) genotypes, but the NaTPI-insensitive (not inhibited by leaf diet) trypsin activity in caterpillars that fed on S++ plants was significantly lower (2.6 TAU) than in those that fed on A plants (4.8 TAU; white bars; F_3,11 = _96.439; P < 0.0001; Fig. 6). These results suggest that NaTPI activity in the leaf diet of the caterpillars did two things: First, it inhibited the NaTPI-sensitive fraction of gut proteinase activity and thereby lowered the trypsin activity per gram of gut tissue. As a result, the fraction of gut trypsin-like activity which is sensitive to additional NaTPI went down from 2.9 TAU in caterpillars that fed on A plants to 0.7 in those that fed on S++ plants. Second, it reduced the NaTPI-insensitive trypsin fraction from 4.8 TAU to 2.6 TAU (white bars; Fig. 6). This is in sharp contrast to what is usually observed: the inhibitor-insensitive fraction tends to be up-regulated in response to the inhibition of the sensitive complement. Our results suggest that NaTPI is a strong inhibitor of *M. sexta* gut trypsin-like activity.

### Stability of NaTPI in relation to gut proteinases

**Figure 7:**
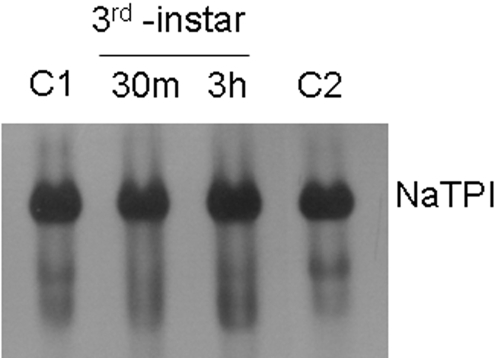
Stability of NaTPI against trypsin of *M. sexta* larvae. Leaf extracts of WT containing NaTPI incubated with 2 trypsin inhibitor units of third-instar gut proteinase for 0.5 h or 3 h at 30°C. Two controls were used: C1 (not incubated) and C2 (incubated without gut proteinases for 3h). Mixtures were resolved on 10% PAGE and then visualized for trypsin inhibitor activity using gel X-ray film contact print technique [60].

In order to test the hypothesis that caterpillars can reduce the inhibitory effects of NaTPI on gut proteinases by proteolytically inactivating PIs [26], NaTPIs containing leaf extracts of WT plants were incubated with gut proteinase from third-instar larvae. Our results showed that NaTPIs were stable in relation to the gut proteinase activity from third-instar *M. sexta* larvae even after 3h of incubation (Fig. 7).

### Changes in NaTPI levels in larval diet elicit rapid responses in gut proteinase activity

**Figure 8:**
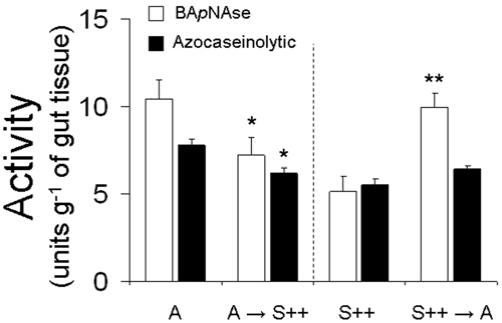
Recovery of inhibited trypsin activity after larvae moved to plants without NaTPI. Gut proteinase activity levels (mean±SEM) in third-instar *M. sexta* larvae that fed on leaves of either A or S++ genotypes. Half of the larvae feeding on either A or S++ genotypes were transferred from either A to S++ genotypes (A→S++) or S++ to A genotypes (S++→A), and after 24 h gut proteinase activity was measured. Total gut proteinase activity was measured using azocasein as a substrate (black bars) and trypsin-like activity was measured using BA*p*NA as a substrate (open bars). Symbols above columns indicate levels of significant differences between either A and A → S++ or S++ and S++→A treatments (*P< 0.05, **P< 0.001, ***P< 0.0001).

To explore whether larvae can alleviate the effects of NaTPI inhibition by rapid changes in gut proteinase activity, we transferred third-instar larvae, half of which had fed on either A or S++ genotypes, to either A or S++ genotypes (A→S++ or S++→A). The gut proteinase activity of larvae transferred after 24 h from A to S++ genotypes was inhibited by NaTPI. While levels of both azocaseinolytic (20%) and BA*p*NAase (30%) activity were reduced after larvae were transferred from A to S++ genotypes (F_3,11-AZOCASEIN = _7.490; P = 0.01; F_3,11-BA*p*NA = _6.918; P = 0.04), larvae transferred from S++ to A genotypes increased BA*p*NAase activity (48%; P = 0.006) but did not significantly increase azocaseinolytic activity (P = 0.08; Fig. 8).

## Discussion

### NaTPI reduces *M. sexta* growth and digestive proteinase activity

By genetically modifying the ability of *N. attenuata* to produce NaTPIs, we were able to determine the effects of NaTPI on the *N. attenuata-M. sexta* plant-insect interaction. Third-instar larvae that fed on genotypes with high levels of NaTPI (WT and S++) attained less mass (38–45%) and experienced lower gut proteinase activity (total proteinase 20–30% and trypsin activity 20–50%) than those that fed on genotypes with low NaTPI (AS-*Natpi* and A) activity levels (Figs. 2, 3, and 6). NaTPI in larval guts decreases proteinase activity and thus the digestibility of plant proteins, which in turn reduces larval mass [12], [15]. However, larvae can respond to high NaTPI levels in the diet by (i) over-producing or down-regulating PI-sensitive proteases [61], (ii) over-producing PI-insensitive proteases [23], [24], (iii) degrading the inhibitors [26], and/or (iv) changing their feeding position on the plant [41]. Caterpillar behavior and within-plant NaTPI heterogeneity allow caterpillars to optimize their growth within the constraints of the digestive duet that occurs between plant and insect [41].

### The digestive duet: how TPI exposure influences the gut proteinase activity of *M. sexta*

*N. attenuata* responds to elicitation not only by increasing NaTPI activity in leaves [40], [41], [43] but also by affecting post-translational changes in the processing of NaTPI subunits; such changes increase the structural diversity of NaTPI isoinhibitors [44]. The results obtained here are consistent with the hypothesis that the post-translational changes in the processing of the NaTPI subunits increase the potential for NaTPI to inhibit gut proteinase activity. NaTPIs extracted from uninduced plants inhibited 20% of the trypsin activity of caterpillars that fed on the WT genotype, whereas NaTPIs extracted from elicited plants inhibited approximately 40% of the trypsin activity (Fig. 5). This is surprising because these extracts were not different in their ability to inhibit bovine trypsin used to determine the concentration of trypsin inhibitor in the extract. These results suggest one of two things: (i) It is possible that caterpillar feeding induced NaTPIs that inhibit trypsin-like *M. sexta* activity, but not bovine trypsin; (ii) Collectively the post-translational changes in the processing of multidomain PIs found in elicited *Nicotiana* plants comprise an adaptive response which enhances this defense. An analysis of the sequence variation revealed that the active domains of PI genes carry the signatures of an evolutionary arms race between plants and their enemies [9], [62]; hence the ability to produce a wide spectrum of structurally and functionally divergent PIs is likely important for PIs' defensive function.

It is well established that insects respond to high dietary PI levels either by producing proteinases of similar substrate specificity that are sterically insensitive to the inhibitor or by degrading the inhibitors [24], [25], [27], [63]. For example, *H. armigera* larvae that ingest high levels of serine PI proteins increase the accumulation of transcripts and proteins of not only midgut serine proteinases (trypsin/chymotrypsin) [21], but also proteinases that are insensitive to the inhibitors that digest the ingested PI proteins [26]. However, in the present study, although *M. sexta* is a natural herbivore of *N. attenuata,* proteinases of larvae were not able to deactivate NaTPIs (Fig. 7); neither were the larvae able to up-regulate NaTPI-insensitive trypsins (Fig. 6). In fact, activity measurements of gut extracts from larvae reared on either A or S++ plants demonstrated that dietary NaTPIs inhibited the inhibitor-sensitive fraction of gut proteinase and, when complemented with NaTPIs extracted from S++ plants, the NaTPI-insensitive (not inhibited by leaf diet) trypsin activity in the caterpillars that fed on S++ plants was almost half (2.6 TAU) of those that fed on A plants (4.8 TAU) (Fig. 6). Although the down-regulation of sensitive proteases can be seen as an adaptation of the insect to save resources, the down-regulation of the NaTPI-insensitive trypsins by *M. sexta* is a surprising finding because it does not improve the insect's fitness. However, although NaTPI inhibited gut proteinase activity in caterpillars 24 h after they were transferred from A to S++ genotypes, caterpillars recovered at least part of their gut proteinase activity when they were transferred from NaTPI-producing host plants to NaTPI-free host plants (Fig. 8). Whatever the mechanism, these rapid changes in gut proteinase activity to dietary changes in NaTPIs suggest that caterpillars can minimize NaTPI inhibitory effects by moving from an elicited leaf with high NaTPI levels to an unelicited leaf [41]. Additional work is needed to characterize the changes in proteolytic activity at the level of transcript accumulation of *Manduca*'s individual gut proteinases. Moreover, since the larvae were derived from a laboratory culture, it will be important to determine whether there exists variation in these responses in natural populations. The results underscore the importance of measuring the effects of plant defenses on herbivores in the context of herbivore feeding behavior on their natural hosts [41], [64].

### Feeding behavior in the context of NaTPI activity and protein content

In both glasshouse and field studies, second- and third-instar larvae have been observed moving from their oviposition sites on basal leaves to leaves at higher stalk positions; leaves at these positions contain low levels of NaTPI per unit total protein [42]–[44]. This change in feeding location can be driven by many different factors, including temperature, predation risk, secondary metabolites, and NaTPI activity [42]–[44]. How plants optimize their PI-based defense allocation may be constrained by the physiology of carbon assimilation. Younger leaves located at higher stalk positions have more protein (RuBisCO) to support their higher photosynthetic rates, which in turn result from the close proximity of fitness sinks (seeds) and sources (leaves) [42]–[44]. Furthermore, when caterpillars feed on leaves at higher stalk positions, they decrease plant fitness [45] and increase their rate of mass gain and level of gut proteinase activity compared to those of larvae that remain on basal leaves (Figs. 2 and 3A). Moreover, extracts of older basal leaves (BOTTOM) inhibited more gut proteinase activity than did extracts of younger stem leaves (MIDDLE) and this differential inhibition was not simply a result of a lower NaTPI concentration in the middle leaves (Fig. 4B). Interestingly, after correcting for concentration differences in the NaTPI fractions between BOTTOM and MIDDLE leaves, BOTTOM leaves inhibited gut proteinase activity about 2.5 times more effectively than did MIDDLE leaves (Fig. 4B), suggesting qualitative rather than quantitative changes in the inhibitor fractions. In addition, the higher protein contents of older leaves on plants grown in 5L rather than 1L pots did not decrease the effects of NaTPI on larval mass (J.A. Zavala, A. Giri and I.T. Baldwin, unpublished data). Our results suggest that i) larval movement alleviates the inhibitory effect of NaTPI on gut proteinase activity, and either ii) qualitative rather than quantitative differences in protein content between leaf positions may alleviate the inhibitory effects of NaTPI on gut proteinase activity, which is consistent with previous studies using artificial diet [30], [65], or iii) other secondary metabolites are involved in the interaction [4], [66], [67].

We conclude that young high-quality leaves with high protein levels alleviated the NaTPI inhibitory effect on gut proteinase activity. In addition, although larvae can tolerate the chronic ingestion of NaTPIs by means of short-term biochemical responses, endogenous NaTPIs of the WT plant represent an effective defense against *M. sexta*: they simply inhibit gut proteinase activity, making the insect unable to adapt to these inhibitors which significantly reduce larval performance.
